# Organic–inorganic hybrid tetrachlorocadmates as promising fluorescent agents for cross-linked polyurethanes: synthesis, crystal structures and extended performance analysis†

**DOI:** 10.1039/d0ra10787e

**Published:** 2021-02-17

**Authors:** Olga Yu. Vassilyeva, Elena A. Buvaylo, Yevheniia V. Lobko, Rostyslav P. Linnik, Vladimir N. Kokozay, Brian W. Skelton

**Affiliations:** Department of Chemistry, Taras Shevchenko National University of Kyiv 64/13 Volodymyrska str. Kyiv 01601 Ukraine vassilyeva@univ.kiev.ua; Department of Surface and Plasma Science, Charles University V Holešovičkách 2 Prague 18000 Czech Republic; School of Molecular Sciences, University of Western Australia M310 Perth WA 6009 Australia

## Abstract

The aim of this work is to apply organic–inorganic hybrid salts made of imidazo[1,5-*a*]pyridinium-based cations and halometallate anions as fluorescent agents to modify cross-linked polyurethane (CPU) for the creation of flexible photoluminescent films. The use of ionic compounds ensures excellent dispersion of the luminescent components in the polymer matrix and prevents solid-state quenching. The absence of phase segregation makes it possible to fabricate uniformly luminescent films with a large area. To this, new tetrachlorocadmate salts [L]_2_[CdCl_4_] (1) and [L′]_2_[CdCl_4_] (2), where L^+^ is 2-methyl-3-(pyridin-2-yl)imidazo[1,5-*a*]pyridinium and [L′]^+^ is 2-methylimidazo[1,5-*a*]pyridinium cations, have been prepared and characterized by IR, NMR, UV-Vis spectroscopy and single crystal X-ray diffraction. The organic cations resulted from the oxidative cyclization-condensation involving CH_3_NH_2_·HCl and 2-pyridinecarbaldehyde in methanol (1), and formaldehyde, CH_3_NH_2_·HCl and 2-pyridinecarbaldehyde in an aqueous media (2). In the crystal of 1, loosely packed tetrahedral cations and π–π stacked anions are arranged in separate columns parallel to the *a*-axis. The pseudo-layered structure of 2 is built of the organic and inorganic layers alternating along the *a* axis. The adjacent CdCl_4_^2−^ anions in the inorganic layer show no connectivity. The organic–inorganic hybrids 1 and 2 were immobilized *in situ* in the cross-linked polyurethane in low content (1 wt%). The photoluminescent properties of 1 and 2 in the solid state and in the polymer films were investigated. The semi-transparent CPU films, that remain stable for months, retain the photoluminescent ability of both hybrids in the blue region with a prominent red shift in their emission.

## Abstract 2

Hybrid salts made of imidazo[1,5-*a*]pyridinium-based cations with easily tunable electronic structures, and halometallate anions that do not include any rare-earth or noble metals were used to fabricate flexible luminescent CPU films.

## Graphical abstract

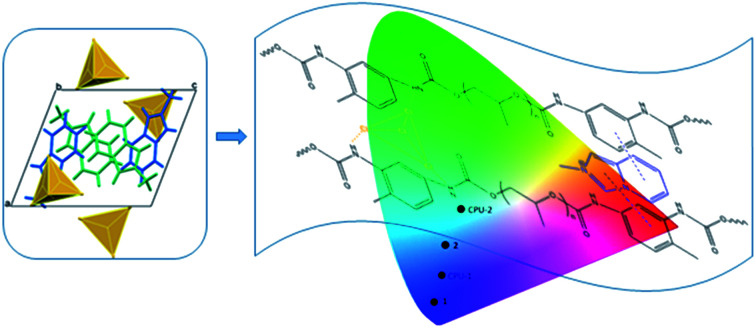

## 1. Introduction

The importance of luminescent materials in modern life can hardly be overestimated. Their growing applications in organic light-emitting diodes (LEDs),^1–3^ solar cells,^4,5^ imaging,^6^ and sensors^7,8^ drive the development of new luminescent compounds. Traditional small organic molecules (organic dyes), semiconductor quantum dots, metal nanoclusters or luminescent transition metal complexes are most widely used as carriers of luminescence. Of the transition metal complexes, those based on heavy second- or third-row transition metals, such as Ru(ii), Rh(i), Ir(iii) or Pt(ii) make the majority of these.^9,10^ These rare metals are quite expensive, and the development of luminescent materials based on less costly and more-abundant transition metals is highly appealing.

There are also problems in the usage of synthetic luminescent compounds, such as luminescence quenching effect,^11,12^ and the impossibility to form the components or coatings with proper forms and mechanical resistance. These drawbacks are eliminated by immobilization of such compounds in a polymer matrix. The isolation of the luminescent molecules by polymer chains can drastically decrease the luminescence quenching effect and, consequently, significantly increase the luminescence efficiency at a noticeable lower content of the luminescent component. Moreover, it is possible to form transparent thin films or other materials with different shapes. Polymer-based luminescent films have received tremendous attention in a wide range of applications such as chemo-/bio-sensing,^13^ solar concentrators,^14^ and down-converters for blue LEDs.^15^

Among the polymers, polyurethane is one of the most versatile plastic materials because of its unique flexibility and pleasant mechanical properties.^16^ Polyurethanes are made by the exothermic reactions between polyols and isocyanates that have more than one reactive isocyanate group (–NCO) per molecule. Since the urethane monomer does not exist, the polymer is almost invariably created during the manufacture of a particular object. The physical properties, as well as the chemical structure of the final polymer, can be tuned by the characteristics of the original reactants and by the way in which the polyurethane ‘building blocks’ are blended.

The imidazo[1,5-*a*]pyridines are an important class of fused nitrogen-containing bicyclic systems due to their biological activity and potential applications in materials chemistry. They also show strong fluorescence intensity and high quantum yield.^17–19^ Encouraged by the successful incorporation of the imidazo[1,5-*a*]pyridinium moiety in the halometallate structure that produced the fluorescent [L]_2_[ZnCl_4_] hybrid salt (L^+^-2-methyl-3-(pyridin-2-yl)imidazo[1,5-*a*]pyridinium cation),^20^ we developed a convenient synthetic strategy to systematically modify the photoluminescent properties of imidazo[1,5-*a*]pyridine species through varying substituents on the polyheterocyclic core as well as through introducing different metal ions.^21^ The next cation in the series, 2-methylimidazo[1,5-*a*]pyridinium, [L′]^+^, has been synthesized by the interaction between equimolar amounts of formaldehyde, methylamine hydrochloride and 2-pyridinecarbaldehyde (2-PCA) in an aqueous solution.

**Scheme 1:**
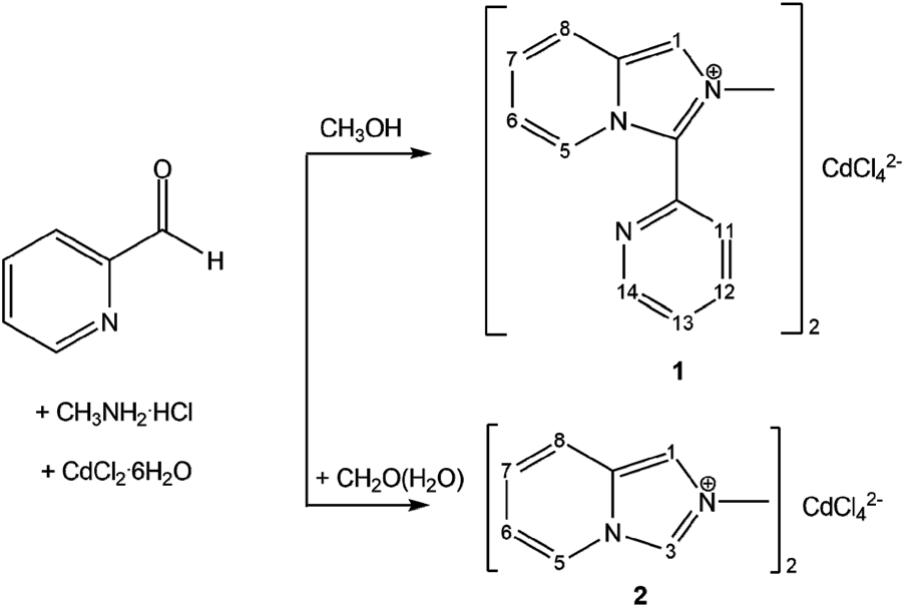
Synthetic schemes for [L]_2_[CdCl_4_] (1) and [L′]_2_[CdCl_4_] (2).

In the present work, the new organic–inorganic hybrids [L]_2_[CdCl_4_] (1) and [L′]_2_[CdCl_4_] (2) have been prepared (Scheme 1) and immobilized in cross-linked polyurethane (CPU) *in situ* in low content (1 wt%). The photoluminescent properties of powdered samples of 1 and 2 in the solid state and in the polymer films were investigated. The semi-transparent CPU films, which remain stable during months, retain the luminescent ability of both hybrids in the blue region with a prominent red shift in their emission.

## 2. Results and discussion

### 2.1 Synthesis and characterization of [L]_2_[CdCl_4_] (1) and [L′]_2_[CdCl_4_] (2)

The substituted imidazo[1,5-*a*]pyridinium cation L^+^ results from the oxidative cyclocondensation between one molecule of methylamine and two molecules of 2-PCA in methanol in the metal-free conditions. The replacement of half the amount of 2-PCA with formaldehyde affords [L′]^+^ in aqueous media. The necessary component of the reaction is acid, which was introduced as a hydrochloride adduct of the amine. The tentative mechanism of the cations formation has been suggested by us before.^20^ Tetrachlorocadmates 1 and 2 were smoothly assembled from the obtained *in situ* monovalent cations, Cd^2+^ ions and chloride anions using an overall CdCl_2_ : CH_2_O : CH_3_NH_2_·HCl : 2-PCA mole ratio of 1 : 0 : 4 : 4 (1) and 1 : 2 : 2 : 2 (2). The hybrids are very soluble in alcohols, *N*,*N*-dimethylformamide (DMF), DMSO and water; they are indefinitely air stable.

The IR spectra of the hybrid salts demonstrate distinctive patterns that were considered characteristic of imidazo[1,5-*a*]pyridinium-based species L^+^ and [L′]^+^ (Fig. S1, see ESI†).^20,21^ Both spectra confirm the presence of alkyl groups and aromatic rings. The spectrum of 2 is dominated by the peak at 802 cm^−1^ ascribed to the out-of-plane C–H bending; two other very intense absorptions are observed at 1148 and 624 cm^−1^. The spectrum is distinguished by the very sharp strong peaks due to aromatic C–H stretching (3120–3020 cm^−1^) and the lack of absorbance in the 1654–1564 and 1542–1452 cm^−1^ regions. The overall richer spectrum of 1 shows a noticeable set of three peaks at 788, 748 and 624 cm^−1^ with no intense band seen around 1148 cm^−1^. The intensity of narrow peaks in the aromatic stretching region (3128–3000 cm^−1^) is also lower.

The room temperature (r.t.) ^1^H NMR spectra of the hybrids in DMSO-*d*_6_ demonstrated the expected sets of signals and correct aromatic/alkyl proton ratios of L^+^ and [L′]^+^ cations for 1 and 2, respectively (Fig. S2 and S3, see ESI;† the numbering diagrams are shown in Scheme 1). CH protons in the five-membered rings are observed as singlets at *δ* = 8.56 (1) [H1] and 9.82 [H3] and 8.24 ppm [H1] (2). The pyridine protons of L^+^ [H5–H8, H11–H14] and [L′]^+^ cations [H5–H8] give eight and four resonances, respectively, between 8.94 and 7.11 ppm. Protons of the CH_3_ groups appear as singlets at 4.30 (1) and 4.27 ppm (2). The close resemblance of the measured spectra with those of other L^+^- and [L′]^+^-containing halometallates^20,21^ suggests conformational stability of the cations in solution irrespectively of the counteranion and thus complete dissociation of the hybrid salts in DMSO.

### 2.2 Structural description of 1 and 2

**Fig. 1:**
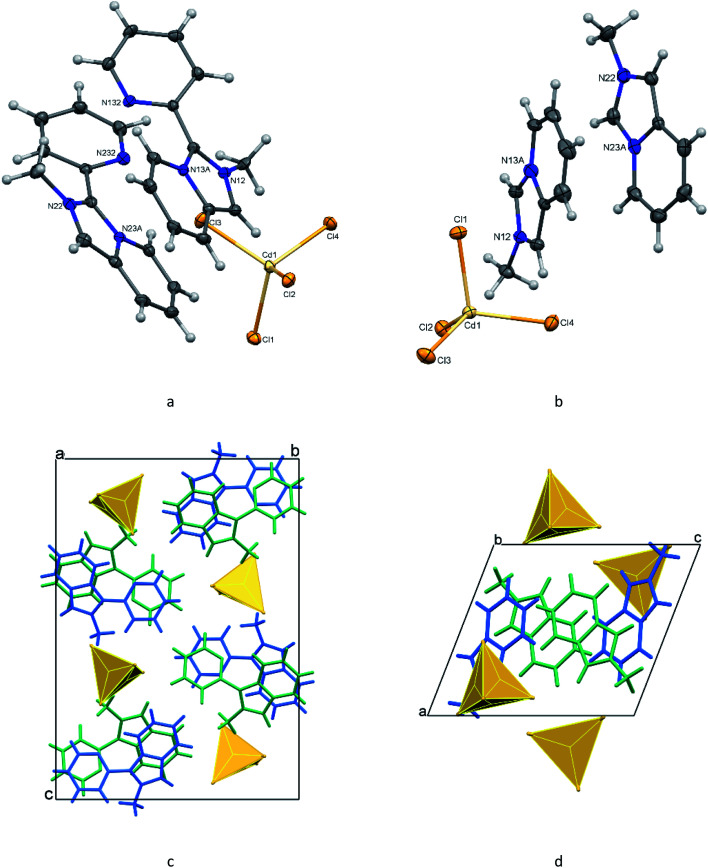
Molecular structures and principal labeling of [L]_2_[CdCl_4_] (1, a) and [L′]_2_[CdCl_4_] (2, b) with displacement ellipsoids drawn at the 50% probability level. Fragments of the crystal packing of 1 (c) and 2 (d) viewed along the *a* and *b* axes, respectively, with the independent cations shown in blue and green.

The organic–inorganic hybrids 1 and 2 are built of discrete 2-methyl-3-(pyridin-2-yl)imidazo[1,5-*a*]pyridinium and methylimidazo[1,5-*a*]pyridinium cations, respectively, and tetrachlorocadmate anions (Fig. 1a and b). In both structures, there are two crystallographically non-equivalent cations (L1 and L2) that do not differ significantly from each other. The bond lengths in the pyridinium rings of the fused cores in L^+^ and [L′]^+^ cations are as expected; the N–C bond distances in the five-membered entities vary from 1.335(2) to 1.405(7) Å. The N atoms in the imidazopyridinium cores are planar with a sum of three angles being equal to 360°. The five- and six-membered rings in the cores are almost coplanar showing dihedral angles between them of less than 2°. In the case of 1, the imidazopyridinium moieties make the dihedral angles of 36.4(2), and 35.9(2)° with the pendant pyridyl rings for cations L1 (N12, N13A) and L2 (N22, N23A), respectively.

The tetrahedral CdCl_4_^2−^ ions in 1 and 2 are slightly distorted with the Cd–Cl distances falling in the range 2.4353(13)–2.4829(13) Å and the Cl–Cd–Cl angles varying from 105.012(17) to 117.283(18)° (Table 1). The maximum differences in the lengths and angles are 0.048 Å, 4.94°, and 0.024 Å, 12.27° for 1 and 2, respectively.

Selected geometric parameters (Å, °) for [L]_2_[CdCl_4_] (1) and [L′]_2_[CdCl_4_] (2)

**Table:** 

1	
Cd1–Cl1	2.4353(13)
Cd1–Cl2	2.4452(13)
Cd1–Cl3	2.4719(13)
Cd1–Cl4	2.4829(13)
Cl1–Cd1–Cl2	108.63(5)
Cl1–Cd1–Cl3	108.48(5)
Cl2–Cd1–Cl3	108.33(4)
Cl1–Cd1–Cl4	113.27(5)
Cl2–Cd1–Cl4	110.92(5)
Cl3–Cd1–Cl4	107.08(5)

**Table:** 

2	
Cd1–Cl1	2.4719(5)
Cd1–Cl2	2.4716(5)
Cd1–Cl3	2.4477(5)
Cd1–Cl4	2.4626(5)
Cl1–Cd1–Cl2	112.106(16)
Cl1–Cd1–Cl3	105.736(17)
Cl2–Cd1–Cl3	110.021(16)
Cl1–Cd1–Cl4	105.012(17)
Cl2–Cd1–Cl4	106.686(17)
Cl3–Cd1–Cl4	117.283(18)

A view of the diagram of the cell contents of 1 (Fig. 1c) shows that cations and anions form separate columns parallel to the *a*-axis. In the column, the alternating L1 and L2 cations are supported by weak aromatic stacking between the offset pyridinium entities of the fused cores with the ring-centroid distances of 3.576 and 4.031 Å. The π–π stacking between the adjacent pendant pyridyl rings of L1 and L2 which are twisted to each other by 19.07°, is also weak [the ring-centroid separations are 3.733 and 3.977 Å]. The loose packing of the identically stacked tetrachlorocadmate anions, with the shortest Cd–Cl⋯Cl–Cd distance being 3.686 Å, leads to a separation of approximately 7.513 Å between the metal atoms in the cation column.

**Fig. 2:**
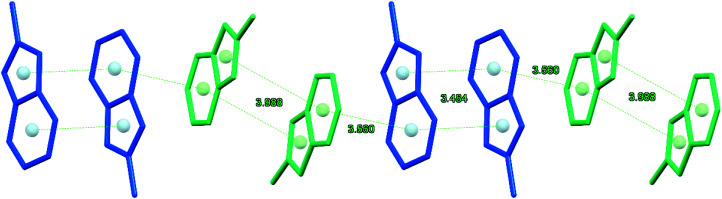
Fragment of the π-bonded chain made of pairs of the inversion-related L1 and L2 cations of [L′]_2_[CdCl_4_] (2).

In the crystal of 2, the organic and inorganic layers alternate along the *a* axis forming a pseudo-layered structure (Fig. 1d). In the organic layer, pairs of [L′]^+^ cations oriented *trans* to each other are arranged in π-bonded chains (Fig. 2). The pairs of cations in the chain demonstrate stronger and weaker 10πe–10πe stacking with the centroid⋯centroid distances for L1 and L2, 3.484 and 3.988 Å, respectively. The adjacent tetrachlorocadmate anions in the inorganic layer show no connectivity; the minimum Cd⋯Cd distance is about 7.754 Å. The consecutive inorganic planes are separated by a distance corresponding to the *a*-axis length (9.4184 Å).

Both 1 and 2 lack classical hydrogen-bonding interactions but possess a variety of C–H⋯Cl–Zn contacts between the organic and inorganic counterparts, a feature common to hybrid chlorometallates with nitrogen-containing aromatic cations.^22,23^ Most of these contacts are longer than the van der Waals contact limit of 2.95 Å and can be considered a result of crystal packing.

### 2.3 Synthesis and characterization of neat CPU and CPU modified with the hybrid salts

**Scheme 2:**
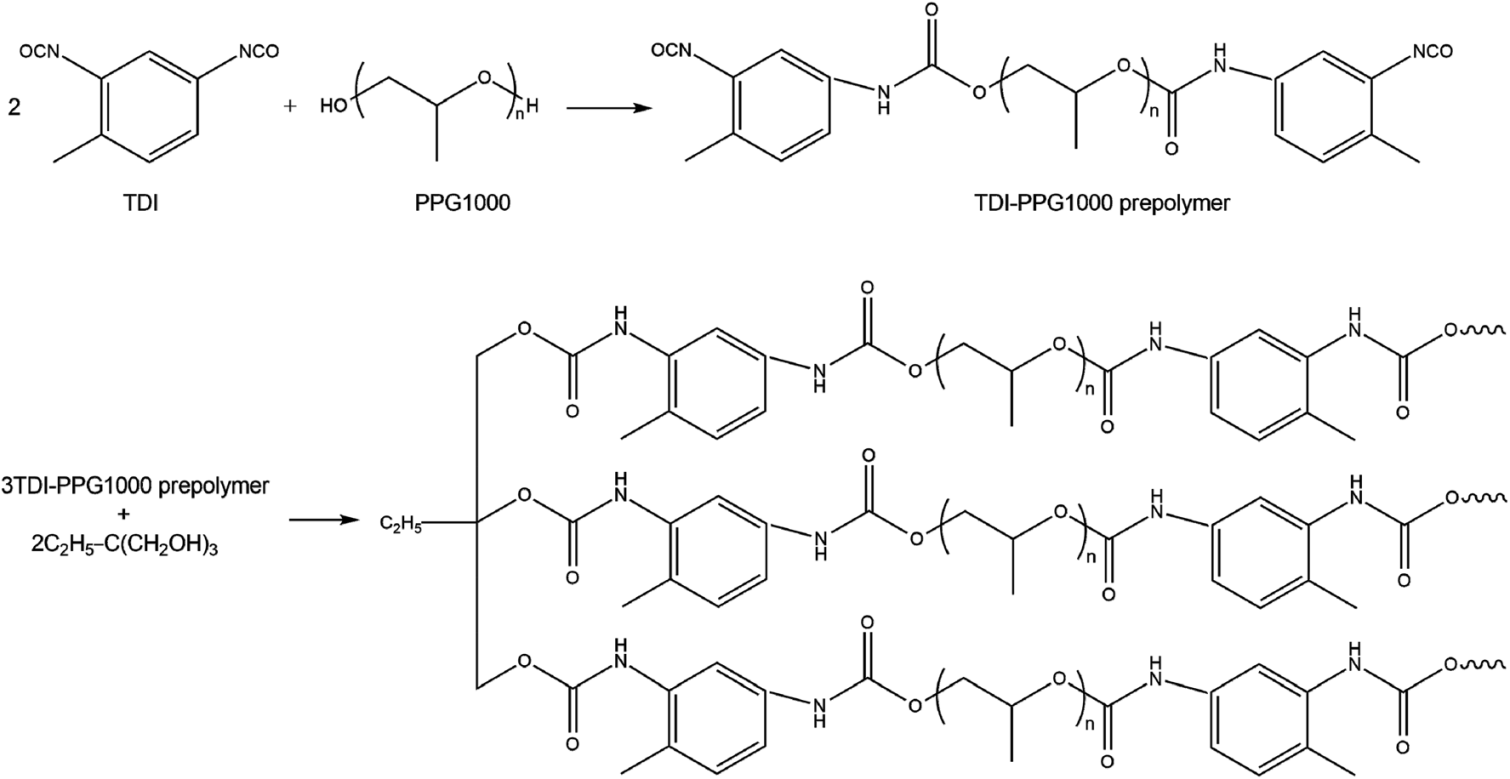
Two-step synthesis of CPU.

A conventional two-step procedure for the preparation of the cross-linked polyurethane is summarized in Scheme 2, where oligomeric polypropylene glycol (PPG1000) reacted with toluene diisocyanate (TDI) in a 1 : 2 mole ratio yielding an NCO-terminated prepolymer, which was further cross-linked with 1,1,1-tris-(hydroxymethyl)-propane (TDP). IR spectroscopy was used to monitor the reaction progress. Characteristic absorption frequencies at 3300, 2972–2868, 1724, 1600–1450 and 1086 cm^−1^ confirmed the presence of –NH, –CH, –C=O, –C=C– and –COC– groups, respectively, in accordance with the proposed structure (Fig. S4, see ESI†). After cooling to r.t., the reaction mass was diluted with DMF to fabricate a film specimen; it was labeled CPU-0.

For the preparation of CPU modified with the hybrid salts, the latter were added to the reaction mass on the cross-linking stage as DMF solutions with the total content of 1 or 2 in the CPU being 1 wt%. The resulting polymers were labeled CPU-1 and CPU-2, respectively. The CPU specimens were prepared by the drop-casting technique as semi-transparent free-standing flexible films of approximately 2 mm thickness. The specimens which are kept at ambient conditions remain mechanically stable during months.

**Fig. 3:**
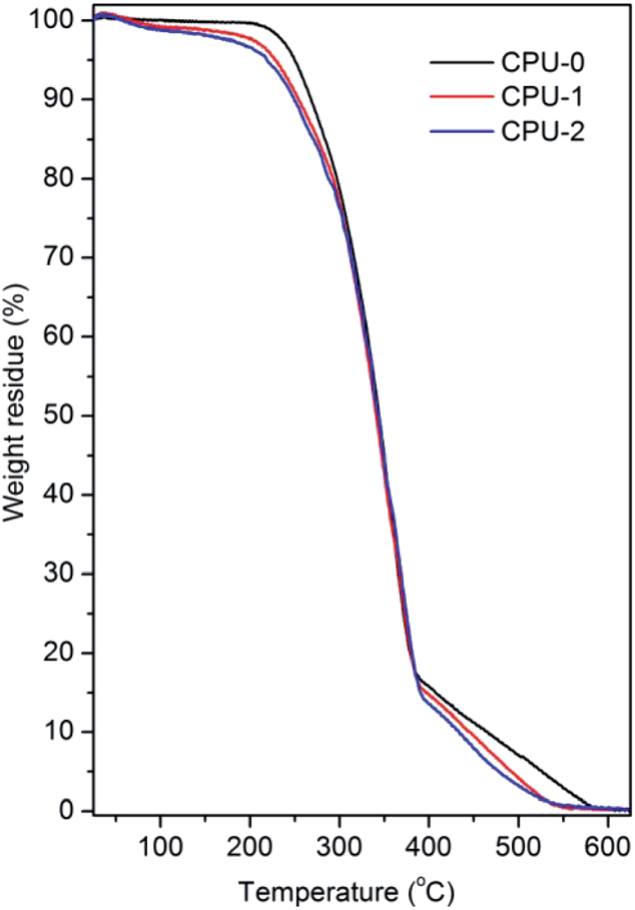
TGA curves of neat CPU-0 and modified with the hybrid salts CPU-1 and CPU-2 at a heating rate of 10 °C min^−1^ under air atmosphere.

Thermal oxidative destruction of CPU-0, CPU-1 and CPU-2 was studied by TGA as depicted in Fig. 3 and summarized in Table 2. The specimens showed a similar thermal behaviour with a slow weight loss above 250 °C under air atmosphere indicative of good thermal stability (Fig. 3). The 5% weight loss at temperatures below 250 °C was due to some trapped volatile components (Table 2). The complete degradation of CPUs was observed above 386 °C with the maximum weight loss of 83%.

**Table:** Thermal oxidative and mechanical parameters of CPU-0, CPU-1 and CPU-2

Sample	5% wt loss *T*, °C	50% wt loss *T*, °C	Max% wt loss *T*, °C	Char residue at 625 °C, %	Tensile strengths, MPa	Elongation at break, %
CPU-0	250	344	386	0	4.3(1)	837(43)
CPU-1	228	341	389	0.30	6.3(2)	350(21)
CPU-2	219	343	393	0.35	8.3(2)	353(23)

**Scheme 3:**
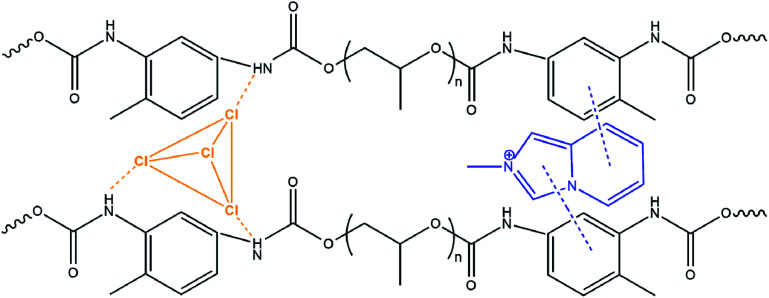
Possible non-covalent interactions between cations and anions of 2 and the polymer matrix in CPU-2.

The incorporation of the hybrid salts in the CPU leads to a significant increase (93% for CPU-2) of the tensile strength of the modified polymer films with simultaneous loss of flexibility (Table 2). An approximately 2.4 times decrease in elongation at break of the CPU film after modification was observed. The enhanced rigidity of the modified polymer films might be attributed to the non-covalent interactions arisen between the cations, anions and polymer matrix such as N–H⋯Cl–Cd charge-supported hydrogen bonds and π–π stacking of aromatic rings, which create additional chemical cross-linking points (Scheme 3).

### 2.4 Photophysical properties of 1, 2, CPU-1 and CPU-2

**Fig. 4:**
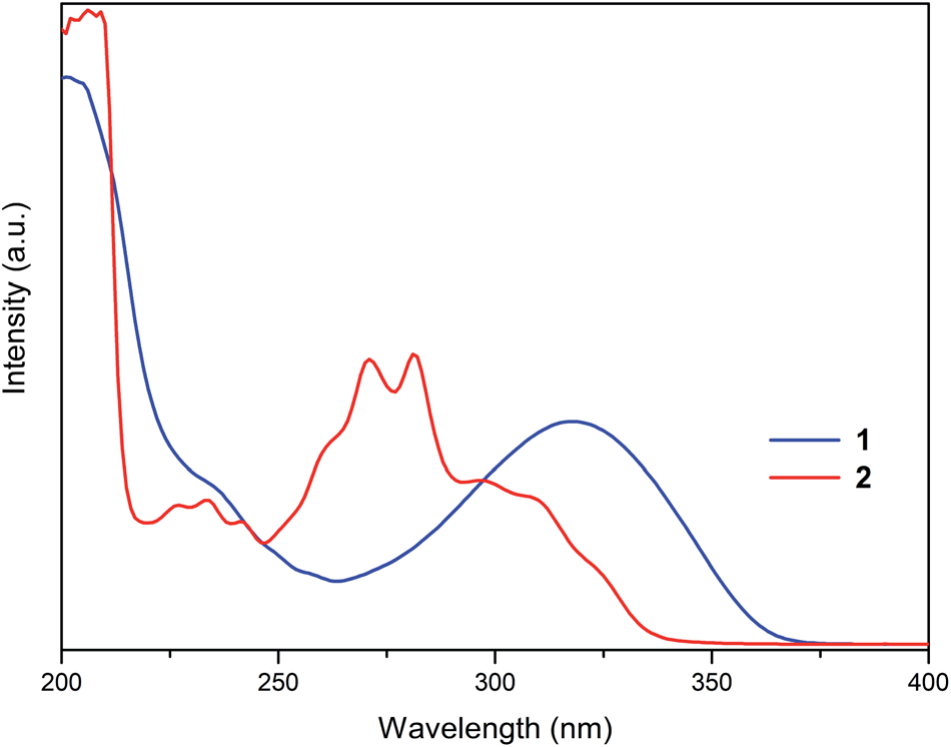
Absorption spectra of 1 (blue) and 2 (red) in CH_3_CN solution (1 × 10^−5^ mol L^−1^) at r.t.

The absorption properties of 1 and 2 were investigated in CH_3_CN solutions (Fig. 4). The colourless solution of 1 exhibited a broad absorption band at 318 nm and a shoulder at 235 nm. The colourless solution of 2 showed a more intricate spectral pattern with dominant peaks at 271 and 281 nm flanked by higher-energy absorption bands in the 226–242 nm region and a shoulder at approximately 300 nm. Given complete dissociation of 1 and 2 in solution established by NMR studies, absorption bands in their UV-Vis spectra were assigned to electronic transitions of the organic cations.

**Fig. 5:**
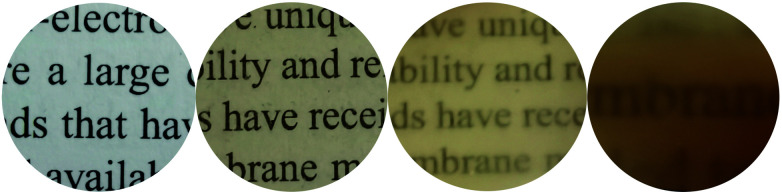
From left to right: photographs of a film-free text, CPU-0, CPU-2 and CPU-1 films with thickness of 2 mm.

**Fig. 6:**
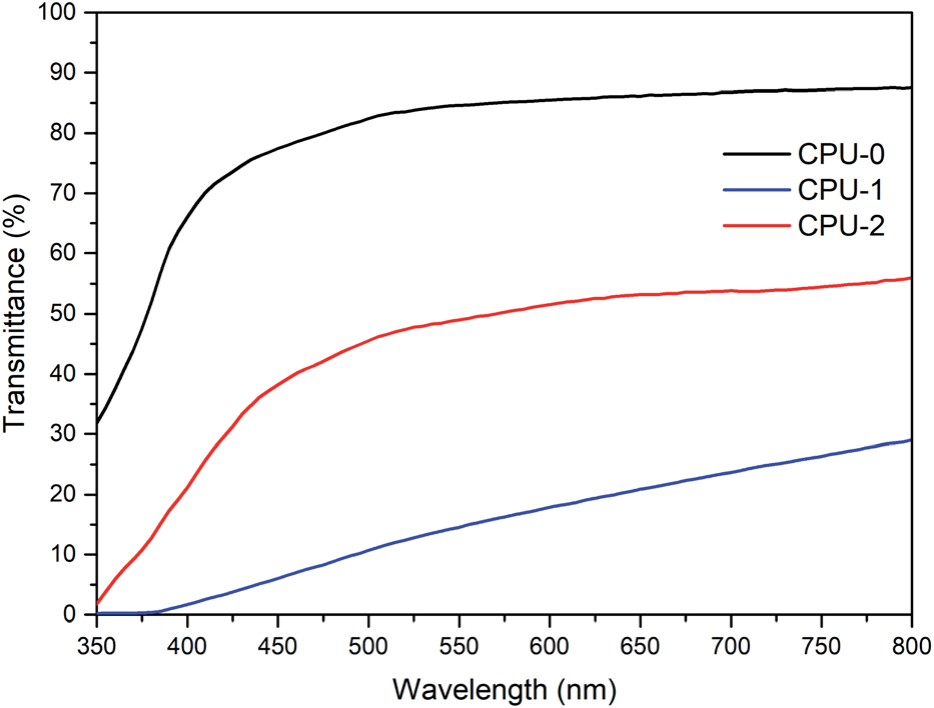
Optical transmittance spectra of CPU-0, CPU-2 and CPU-1 films with thickness of 2 mm.

The incorporation of the hybrid salts in CPU affected both coloration of the resulting films and their optical performance. The films photographs and optical transmittance spectra are depicted in Fig. 5 and 6, respectively. Comparing CPU-0, CPU-1 and CPU-2, CPU-1 film with deeper yellow appearance showed a higher cutoff wavelength of 364 nm and significantly lower transmittance of 29% at the wavelength of 800 nm. It could be related to the presence of an additional aromatic group in the structure of 1.

**Fig. 7:**
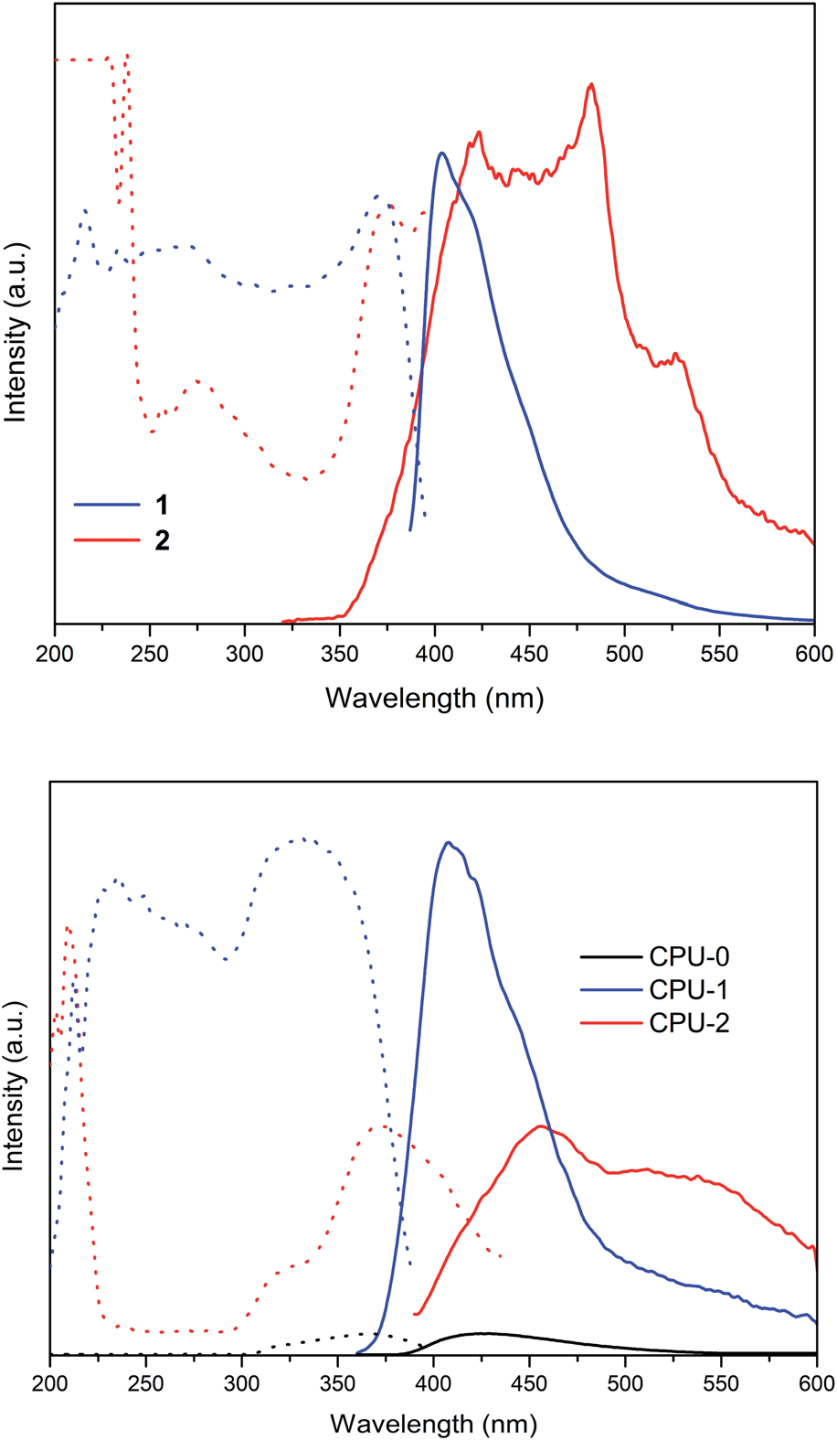
The excitation (dotted) and emission spectra (solid) of powdered hybrid salts 1 and 2 (top) and CPU-0, CPU-1 and CPU-2 films (bottom) at r.t.

**Fig. 8:**
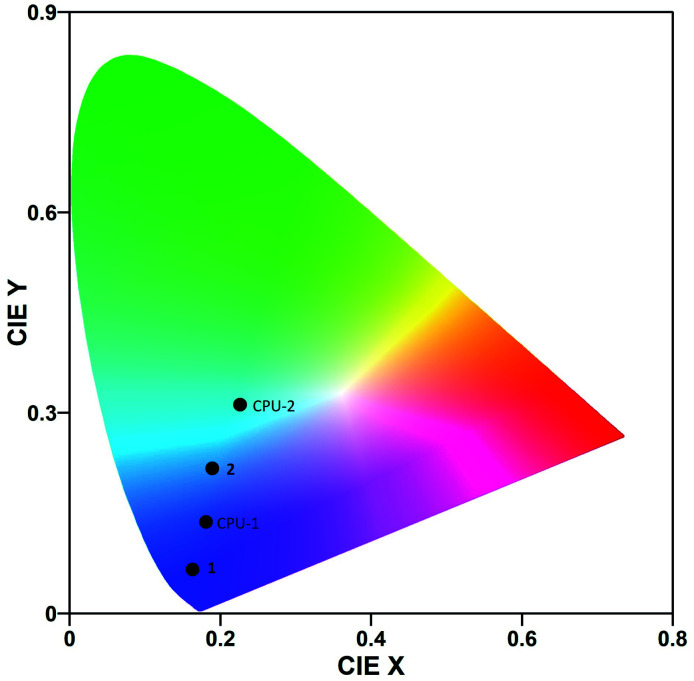
CIE 1931 chromaticity coordinates of 1 and 2 in the solid state and in CPU films.

The photoluminescence spectra of the crystalline powder samples of 1 and 2 differ in shapes and maxima of emission (Fig. 7 top, Table 3). Hybrid salt 1 excited at 370 nm shows a broad intense unsymmetrical band with maximum at 404 nm and a full width at half maximum of 51 nm, while the much broader intense emission of 2 (*λ*_ex_ = 376 nm) spans the 350–600 nm region with a dominating peak at 482 nm (Fig. 7 top). The CIE 1931 chromaticity diagram of 1 and 2 in the solid state (Fig. 8) reflects the difference in their photoluminescence colours with the emission of 1 lying to the deep-blue side of the visible spectrum with CIE of (0.16, 0.07) and the emission of 2 entering the sky-blue region with CIE of (0.19, 0.22).

**Table:** Parameters of the photoluminescence spectra of powdered hybrid salts 1 and 2, and CPU-0, CPU-1 and CPU-2 films

Sample	*λ*_ex_, nm	*λ*_lum_, nm	Stocks shift, nm
CPU-0	365	426	61
1 (powder)	370	404	34
		420 (shoulder)	
		450 (shoulder)	
CPU-1	334	408	74
		422 (shoulder)	
		446 (shoulder)	
2 (powder)	376	423	47
		482	
		526	
CPU-2	370	456	50
		525	

Since the hybrid salts 1 and 2 differ only by the presence of a pendant pyridyl substituent in L^+^ (1), their nonsimilar spectral patterns are clearly related to the difference in the electronic structures of the organic cations, L^+^ and [L′]^+^. The additional pyridyl ring in 1 brings an effect of narrowing the emission line and decreasing the Stocks shift for 1 by 13 nm compared to that for 2. Also, the crystal packing arrangements that vary for 1 and 2 (separated cation and anions stacks *vs.* a layered structure, respectively) might stronger contribute to the emission output in the latter case.

Using quinine sulfate as a reference, the fluorescence quantum yields (*Φ*_F_) of 1 and 2 in CH_3_CN were measured to be 0.29 and 0.10, respectively. Thus, when a π-conjugating substituent is present at C-3, *Φ*_F_ is almost 3-fold enhanced. A great influence of π-conjugating substituents on absorption and emission maxima and *Φ*_F_ of neutral 2-azaindolizines (imidazo[1,5-*a*]pyridines) was observed by F. Shibahara *et al.*^24^ Another study established *Φ*_F_ improvement due to the salt formation of the initial imidazo[1,5-*a*]pyridine precursor.^17*c*^

The neat CPU-0 specimen showed negligible photoluminescence. The photoluminescence spectrum of 1 in a CPU matrix mostly keeps its pattern and maximum of emission (Fig. 7 bottom, Table 3). A CPU-1 specimen excited at 334 nm exhibited a broad intense unsymmetrical band with maximum at 408 nm and a full width at half maximum of 67 nm that corresponds to blue-light emission with CIE of (0.18, 0.14). Similarly, the intense emission of 2 in a CPU matrix (*λ*_ex_ = 370 nm) still covers the 380–600 nm region but now is peaked at 456 nm with an obvious change of the spectrum shape. The emission colour of 2 thus shifts to the bluish-green region with CIE of (0.23, 0.31).

These observations suggest closer interaction of [L′]^+^ cations of 2 with polymer macromolecules owing to their compact size so that changes in photoluminescence behaviour of 2 in a CPU matrix are more pronounced. This idea also correlates with the tensile strength parameter for 2 being a slightly higher compared to that for 1 (Table 2). A cancelled influence of the crystal packing upon dispersion of 2 in CPU could be considered as well.

## 3. Conclusion

Two new organic–inorganic hybrid salts [L]_2_[CdCl_4_] (1) and [L′]_2_[CdCl_4_] (2), where L^+^ is 2-methyl-3-(pyridin-2-yl)imidazo[1,5-*a*]pyridinium and [L′]^+^ is 2-methylimidazo[1,5-*a*]pyridinium cations, have been prepared and characterized by IR, NMR, UV-Vis spectroscopy and single crystal X-ray diffraction. The organic cations are products of the oxidative cyclization-condensation involving CH_3_NH_2_·HCl and 2-PCA in methanol (1), and formaldehyde, CH_3_NH_2_·HCl and 2-PCA in water media (2). In the crystal of 1, loosely packed tetrahedral cations and π–π stacked anions are arranged in separate columns parallel to the *a*-axis. The pseudo-layered structure of 2 is built from alternating organic and inorganic layers with adjacent CdCl_4_^2−^ anions in the inorganic layer showing no connectivity.

The incorporation of the hybrid salts in CPU in low content (1 wt%) did not affect thermal stability of the resulting CPU-1 and CPU-2 but significantly increased tensile strength of the free-standing polymer films (93% for CPU-2). The enhanced rigidity of the modified CPUs was explained by the appearance of non-covalent interactions that created additional cross-linking points. The semi-transparent CPU films remained stable during months.

The photoluminescent properties of 1 and 2 in the solid state and in CPU films were compared. Varying emission colours observed for the hybrids in the blue region reflect the differences in the electronic structures of their organic cations and packing arrangements. The modified CPUs retain the photoluminescent ability of both hybrid salts with a prominent red shift in their emission.

Applying ionic compounds as fluorescent agents to modify cross-linked polyurethane ensures excellent dispersion of the luminescent species in the polymer matrix and avoids phase segregation so that fabrication of uniformly luminescent films of a large area is made possible. The organic–inorganic hybrid salts made of imidazo[1,5-*a*]pyridinium-based cations with easily tunable electronic structures, and halometallate anions that do not include any rare-earth or noble metals, are promising components of luminescent CPU films for flexible optoelectronic devices.

## 4. Experimental

### 4.1 Materials and general methods

For the synthesis of hybrid salts, 2-PCA (Merck) was used as received; all other chemicals were purchased from local suppliers and used without further purification. All solvents were of AP-grade; all the experiments were carried out in air. For the preparation of cross-linked polyurethanes, polypropylene glycol with a molecular weight of 1000 (PPG1000, Merck) was dried under pressure of 300 Pa at 120 °C for 3 hours. TDI (2,4-/2,6-isomers = 80/20) and DMF were purchased from Sigma and distilled under vacuum. TMP (98%, Sigma) was dried under vacuum at 40–42 °C.

Elemental analyses for C, H, N and Cl for 1 and 2 were performed with a Perkin-Elmer 2400 analyzer. The ^1^H NMR spectra of 1 and 2 in DMSO-*d*_6_ were measured using a Mercury 400 Varian spectrometer at 400 MHz at r.t. The chemical shifts (*δ*) values are given in ppm downfield from internal Me_4_Si. *J* values are in Hertz. IR spectra of the hybrid salts were recorded on a Perkin-Elmer 1600 FT-IR instrument from KBr pellets in the 400–4000 cm^−1^ region. The attenuated total reflection Fourier transform infrared (ATR/FTIR) spectrum of neat CPU-0 was recorded on a Tensor 37 (Bruker, USA) instrument in the 650–4000 cm^−1^ region. The absorption spectra of 1 and 2 in CH_3_CN were measured between 200 and 800 nm on a Shimadzu UV-2401PC UV-Vis recording spectrophotometer at r.t.

Optical transmittance of CPU-0, CPU-1 and CPU-2 films was characterized by a Multiscan GO spectrophotometer (Thermo Scientific). Thermal destruction of the films was investigated in the 25–650 °C temperature range in air with a Derivatograph Q-1500D (Paulik, Paulik and Erdey system). Measurements of tensile strength were performed on the film specimens in the form of spatula with the gauge length 25 mm and the thickness of 2 mm using 1925 RA-10 M Testing System (Uralpromtek, Russia) at a stretching speed of 40 mm min^−1^ under a 0.5 kN load.

Fluorescence spectra of powdered samples of 1 and 2, as well as the CPU films, were measured with a Perkin Elmer Spectrometer LS 55 equipped with a xenon flash lamp at r.t. The excitation and emission spectra were recorded in optimal conditions at maximum wavelengths for each investigated system in the range 200–800 nm. The Comission Internationale de l’Eclairage (CIE 1931) coordinates values of 1, 2, CPU-1 and CPU-2 were calculated using their photoluminescence spectra values plotted through GoCIE V2 software.^25^ The fluorescence quantum yields of 1 and 2 were determined by comparing the integrated fluorescence intensities and the absorbance values of the hybrid salts in CH_3_CN (excited at 335 nm) using acidified quinine sulfate (*Φ*_F_ = 0.55, water), which is a common photoluminescence quantum yield standard for characterizing blue emitters.^26^

#### 4.1.1 Synthesis of [L]_2_[CdCl_4_] (1)

2-PCA (0.38 ml, 4 mmol) was added dropwise to CH_3_NH_2_·HCl (0.27 g, 4 mmol) dissolved in 10 ml methanol in a 50 ml conical flask. After stirring for half an hour at r.t., the resultant yellow solution was left in open air overnight and gradually turned olive. Then, solid CdCl_2_·2.5H_2_O (0.23 g, 1 mmol) was added to the flask and the reaction mixture was stirred for 40 min at 30 °C, filtered and left to evaporate. Subsequently colourless shiny needles of 1 suitable for X-ray analysis were formed. The crystals were filtered off, washed with diethyl ether and dried in air. More product was obtained upon slow evaporation in air of the mother liquor. Yield: 81% (based on cadmium). FT-IR (*ν*, cm^−1^): 3440br, 3128, 3110, 3080, 3060, 3010, 2954, 2924, 2854, 1654, 1586, 1514, 1474, 1426, 1372, 1332, 1298, 1252, 1178, 1152, 1104, 1040, 992, 946, 840, 788vs, 748, 664, 612, 570, 558, 500, 434, 408. ^1^H NMR (400 MHz, DMSO-*d*_6_): *δ* (ppm) 8.93 (d, 1H, *J* = 4.4 Hz, H14), 8.72 (d, 1H, *J* = 7.3 Hz, H5), 8.56 (s, 1H, H1), 8.24–8.16 (m, 2H, H11 + H12), 8.03 (d, 1H, *J* = 9.3 Hz, H8), 7.76–7.74 (m, 1H, H13), 7.38 (t, 1H, *J* = 8.1 Hz, H7), 7.24 (t, 1H, *J* = 6.8 Hz, H6), 4.30 (s, 3H, CH_3_). Anal. calcd for C_26_H_24_Cl_4_N_6_Cd (674.73): C 46.28; H 3.59; N 12.46; Cl 21.02%. Found: C 46.44; H 3.43; N 12.58; Cl 20.91%.

#### 4.1.2 Synthesis of [L′]_2_[CdCl_4_] (2)

Paraform (0.13 g, 4.5 mmol) was dissolved in boiling water (10 ml) in a 50 ml conical flask and left to cool at r.t. Solid CH_3_NH_2_·HCl (0.27 g, 4 mmol) was then added to the flask and the solution was stirred vigorously for an hour at r.t. After that it was filtered and left in open air overnight. 2-PCA (0.38 ml, 4 mmol) was added next day followed by solid CdCl_2_·2.5H_2_O (0.23 g, 1 mmol) and the solution was stirred for 30 min at r.t., then filtered and allowed to evaporate. Colourless plate-like crystals of 2 suitable for X-ray crystallography were formed within a few days. The crystals were filtered off, washed with diethyl ether and dried in air. Yield: 87% (based on cadmium). FT–IR (*ν*, cm^−1^): 3428br, 3120s, 3094s, 3050s, 3020, 2956, 2920, 1654, 1564, 1542, 1452, 1374, 1352, 1328, 1258, 1220, 1148vs, 1038, 920, 802vs, 764, 742, 624s, 500, 434, 410. ^1^H NMR (400 MHz, DMSO-*d*_6_): *δ* (ppm) 9.82 (s, 1H, H3), 8.68 (d, 1H, *J* = 6.4 Hz, H5), 8.24 (s, 1H, H1), 7.80 (d, 1H, *J* = 9.3 Hz, H8), 7.23 (t, 1H, *J* = 7.8 Hz, H6), 7.11 (t, 1H, *J* = 6.8 Hz, H7), 4.27 (s, 3H, CH_3_). Anal. calcd for C_16_H_18_Cl_4_N_4_Cd (520.56): C 36.92; H 3.49; N 10.76; Cl 27.24%. Found: C 37.22; H 3.38; N 10.98; Cl 27.01%.

### 4.2 Single crystal structure determination of 1 and 2

Crystallographic data for the structures were collected at 100(2) K on an Oxford Diffraction Gemini diffractometer using Mo-Kα (*λ* = 0.71073 Å) radiation. Following analytical absorption corrections and solution by direct methods, the structures were refined against *F*^2^ with full-matrix least-squares using the program SHELXL-2017.^27^ All hydrogen atoms were added at calculated positions and refined by use of a riding model with isotropic displacement parameters based on those of the parent atom. Anisotropic displacement parameters were employed for the non-hydrogen atoms. Details of the data collection and processing, structure solution and refinement for 1 and 2 are summarized in Table 4 while the selected bond lengths and angles data are presented in Table 1.

**Table:** Crystallographic parameters and refinement data for 1 and 2

Compound	1	2
Empirical formula	C_26_H_24_CdCl_4_N_6_	C_16_H_18_CdCl_4_N_4_
Formula weight	674.71	520.54
Crystal system	Monoclinic	Triclinic
Space group	*P*2_1_/*c*	*P*1̄
*a*/Å	7.5133(2)	9.4184(3)
*b*/Å	15.9736(6)	10.7646(4)
*c*/Å	22.5132(8)	10.7332(4)
*α*/°	90	99.055(3)
*β*/°	94.998(3)	110.834(4)
*γ*/°	90	90.861(3)
*V*/Å^3^	2691.64(16)	1001.39(7)
*Z*	4	2
*D*_cal_/g cm^−3^	1.665	1.726
*μ*/mm^−1^	1.237	1.631
Reflections collected	29 999	38 485
Unique reflections	8604	11 183
*R*_int_	0.0612	0.0423
Data/restraints/parameters	8604/0/337	11183/0/228
GOF	1.045	1.033
*R*_1_ [*I* > 2*σ*(*I*)]	0.0627	0.0375
w*R*_2_ [*I* > 2*σ*(*I*)]	0.1328	0.0761
*R*_1_ (all data)	0.0788	0.0560
w*R*_2_ (all data)	0.1411	0.0847
Δ*ρ*_max/min_/e Å^−3^	1.942 and −0.8	1.603 and −0.796
CCDC	1958965	1959131

### 4.3 Synthesis of CPU and fabrication of films specimens

Cross-linked polyurethane was prepared in a conventional two-step procedure. First, an isocyanate-terminated prepolymer containing 5.9 wt% free NCO (wt% NCO_theor._ = 6.2%) was synthesized from PPG1000 (25 g, 25 mmol) and TDI (8.71 g, 50 mmol). The reaction was carried out in a 50 ml three-neck round-bottom flask at 120 °C for 1.5 hours under magnetic stirring in a dried argon atmosphere. The progress was monitored by IR-spectroscopy. At the second step, the prepolymer (6 g, 4.29 mmol) was cross-linked with TMP (0.38 g, 2.68 mmol) at 70–75 °C in an oil bath with 5 min stirring under a dried argon atmosphere.

The reaction mass was then cooled to r.t., diluted with DMF (3 mL), mixed thoroughly for 15 min and poured into a Teflon mold (10 cm × 6.5 cm × 5 cm). To ensure reaction completion and removal of residual solvent, the mold was cured in a conventional oven at 65 °C for 10 hours with subsequent vacuuming at 65 °C to a constant weight. The resulting specimen labeled CPU-0 as a film of approximately 2 mm thickness was easily detached from the Teflon surface and stored in air. Yield: 6.28 g. IR (ATR, neat, cm^−1^): 3300, 2972, 2929, 2897, 2869, 1726, 1600, 1533, 1506, 1450, 1370, 1224, 1080, 1016, 927, 870, 816, 767.

For the preparation of CPU modified with the hybrid salts, the latter were added to the cross-linked polyurethane mass as DMF solutions with the total content of 1 and 2 in the CPU being 1 wt%. The dispersions were treated in the same way as for CPU-0 to yield films of CPU-1 and CPU-2, respectively.

## Authors contribution

Study concept and design: O. Y. Vassilyeva; acquisition of data: E. A. Buvaylo and Y. V. Lobko (synthesis), B. W. Skelton (X-ray crystallography), Y. V. Lobko (CPU characterization), R. P. Linnik (fluorescence spectra); analysis and interpretation of data: O. Y. Vassilyeva (synthetic, structural and spectral data) and Y. V. Lobko (CPU data); drafting and revising of the manuscript: O. Y. Vassilyeva and Y. V. Lobko; study supervision: V. N. Kokozay.

## Conflicts of interest

There are no conflicts to declare.

## Supplementary Material

RA-011-D0RA10787E-s001

RA-011-D0RA10787E-s002
