# Supplementary material for: Organic–inorganic hybrid tetrachlorocadmates as promising fluorescent agents for cross-linked polyurethanes: synthesis, crystal structures and extended performance analysis
Source: RSC Adv. 2021 Feb 17;11(13):7713–22. doi: 10.1039/d0ra10787e (PMC8695196; doi:10.1039/d0ra10787e)
Supplement: RA-011-D0RA10787E-s001 [file RA-011-D0RA10787E-s001.pdf]

Electronic Supplementary Material (ESI) for RSC Advances.
This journal is © The Royal Society of Chemistry 2021

Organic-inorganic hybrid tetrachlorocadmates as promising fluorescent agents for cross-linked polyurethanes: synthesis, crystal structures and extended performance analysis

Olga Yu. Vassilyeva, Elena A. Buvaylo, Yevheniia V. Lobko, Rostyslav P. Linnik, Vladimir N. Kokozay, and Brian W. Skelton

Supplementary Information

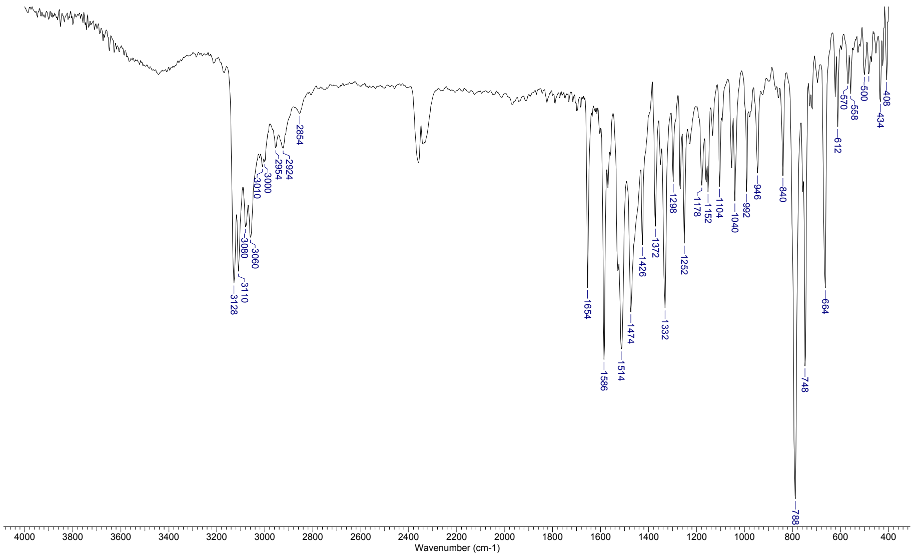

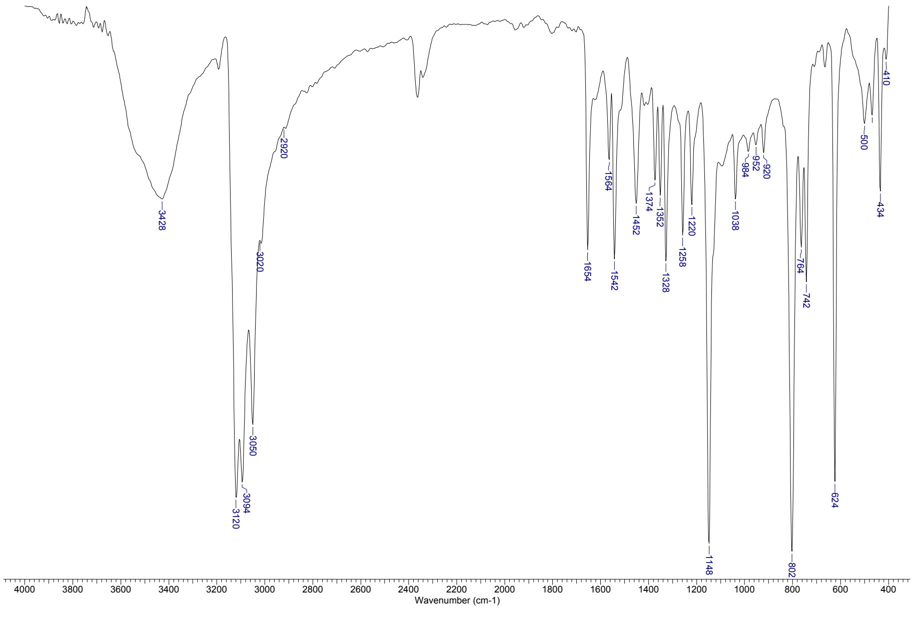

Figure S1. IR spectra of $[L]_2[CdCl_4]$ (**1** top) and $[L']_2[CdCl_4]$ (**2**, bottom).

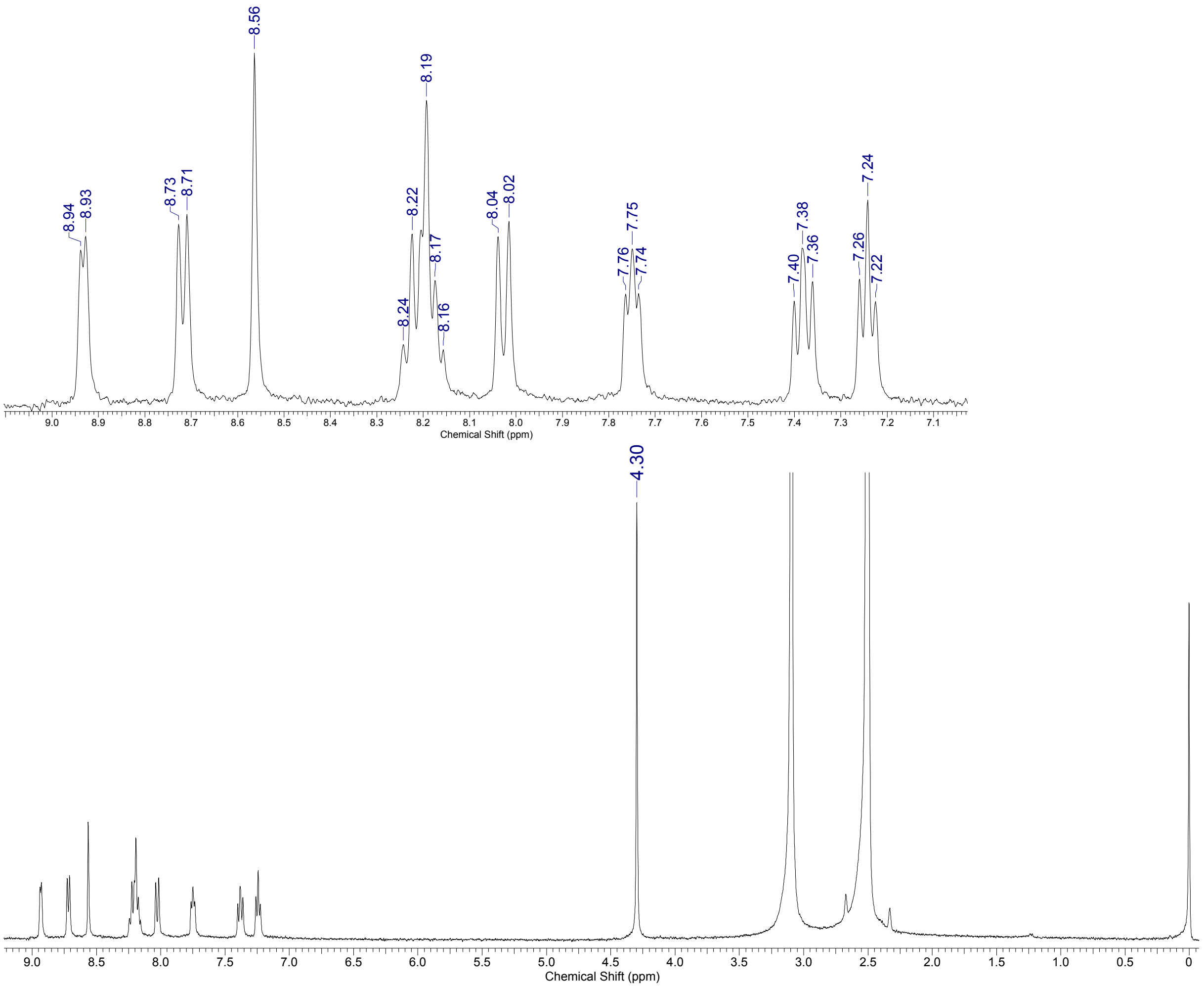

Figure S2. 400 MHz ^{1}HNMR spectrum of $[L]_2[CdCl_4]$ (**1**) in DMSO-d_6 at 293 K in the ranges 9–7 ppm (top) and 9–0 ppm (bottom).

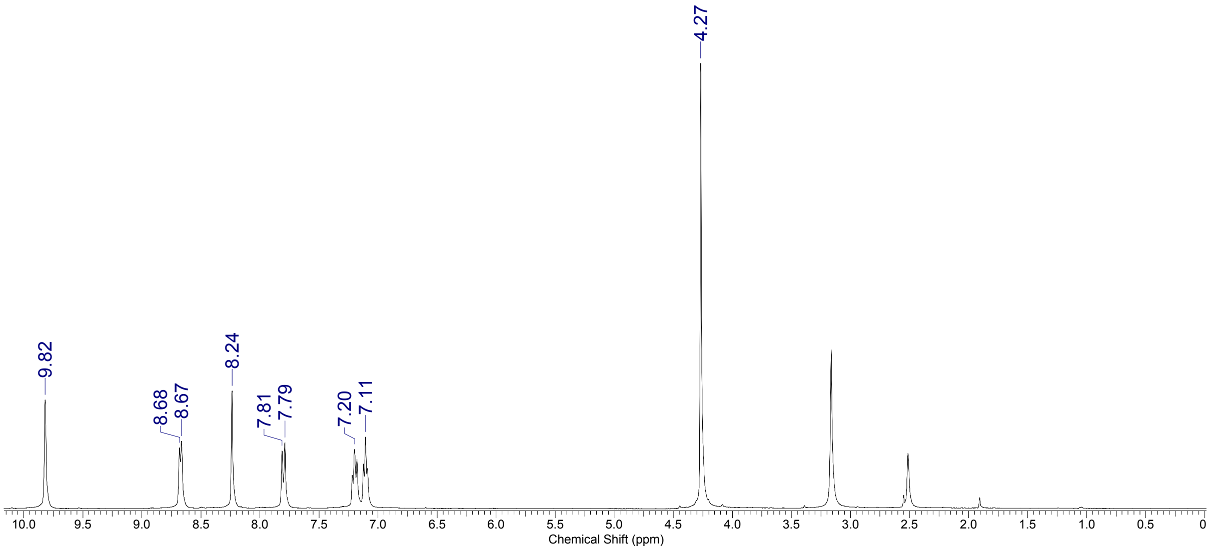

Figure S3. 400 MHz ^{1}HNMR spectrum of $[L']_2[CdCl_4]$ (**2**) in DMSO-d_6 at 293 K between 10 and 0 ppm.

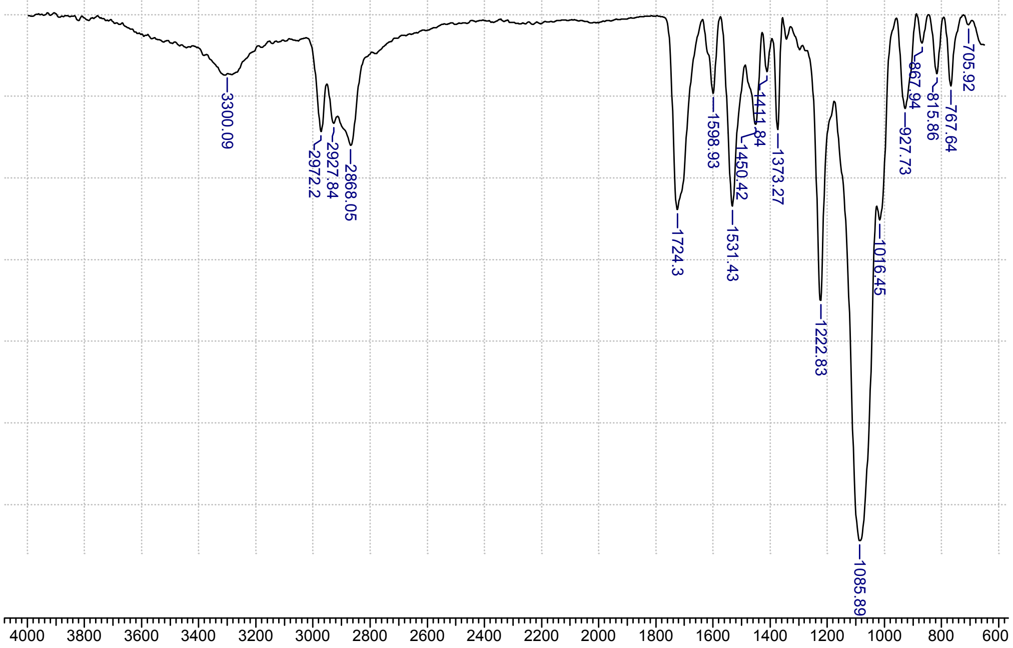

Figure S4. IR spectrum of the neat cross-linked polyurethane.
